# Automatic Detection of Real Damage in Operating Tie-Rods

**DOI:** 10.3390/s22041370

**Published:** 2022-02-10

**Authors:** Francescantonio Lucà, Stefano Manzoni, Alfredo Cigada, Silvia Barella, Andrea Gruttadauria, Francesco Cerutti

**Affiliations:** Politecnico di Milano-Department of Mechanical Engineering, Via La Masa, 20156 Milan, Italy; stefano.manzoni@polimi.it (S.M.); alfredo.cigada@polimi.it (A.C.); silvia.barella@polimi.it (S.B.); andrea.gruttadauria@polimi.it (A.G.); francesco4.cerutti@mail.polimi.it (F.C.)

**Keywords:** automatic damage detection, tie-rod, structural health monitoring, continuous monitoring, real damage, statistical pattern recognition, operational modal analysis

## Abstract

Many researchers have proposed vibration-based damage-detection approaches for continuous structural health monitoring. Translation to real applications is not always straightforward because the proposed methods have mostly been developed and validated in controlled environments, and they have not proven to be effective in detecting real damage when considering real scenarios in which environmental and operational variations are not controlled. This work was aimed to develop a fully-automated strategy to detect damage in operating tie-rods that only requires one sensor and that can be carried out without knowledge of physical variables, e.g., the axial load. This strategy was created by defining a damage feature based on tie-rod eigenfrequencies and developing a data-cleansing strategy that could significantly improve performance of outlier detection based on the Mahalanobis squared distance in real applications. Additionally, the majority of damage-detection algorithms presented in the literature related to structural health monitoring were validated in controlled environments considering simulated damage conditions. On the contrary, the approach proposed in this paper was shown to allow for the early detection of real damage associated with a corrosion attack under the effects of an intentionally uncontrolled environment.

## 1. Introduction

The development of automatic damage-detection strategies has played a key role in the condition-based maintenance of mechanical, civil and aerospace structures [1,2]. Vibration-based damage identification approaches have been widely adopted for long-term continuous monitoring [3,4]. The fundamental idea of these approaches is that damage-induced changes in physical properties (mass, damping and stiffness) are reflected in changes in modal parameters (eigenfrequencies, modal damping and mode shapes) [5,6]. According to this principle, modal parameters can be adopted to describe the state of health of a monitored structure and thus be used to define effective damage features.

A limitation of modal-based damage-detection strategies is that structural and physical properties are also dependent on environmental and operational conditions and not only on damage [7,8]. Furthermore, environmental and operational variations often cause changes in vibration properties that are greater than those caused by damage [9]. Many works in the literature have suggested strategies to overcome this limitation [10,11,12,13], but there have been few applications to structures under real operating conditions, especially when damage is present. The authors of this work propose an automatic algorithm that can be used for the structural health monitoring of beam-like structures; this algorithm was validated with a one-of-a-kind application where real damage was detected on full-scale structural elements under the effects of an uncontrolled environment. 

More specifically, the test case was represented by tie-rods, i.e., tensioned slender beams widely adopted in civil structures to balance the lateral forces of arches and vaults (though the proposed strategy can work for any kind of tensioned beam, such as the ties and struts of space frames or the diagonal braces of a truss girder). Despite their simple geometry, the interpretation of tie-rod modal parameters is complex due to many different physical variables (e.g., axial load and constraints characteristics) that cause changes in the structural properties under operating conditions [14]. Moreover, the above-mentioned physical variables are generally affected by significant uncertainty when real tie-rods are considered.

A review of the state of the art on the topic of vibration-based tie-rod monitoring revealed that the attention of the researchers has been mainly focused on the identification of the axial load as a mean to assess the state of health of structures (the arch or vault where the tie-rod is adopted) [15]. Indeed, the only possible direct measurement of tie-rod tension must be obtained through the adoption of strain gauges calibrated during a tensioning procedure, which is not a viable solution when operating tie-rods are considered. Thus, many research activities have been devoted to the development of indirect approaches to estimate the axial load from vibration measurements (some examples can be found in [16,17,18,19,20,21,22]). 

A common aspect of the above-mentioned works is that none of them considered that a tie-rod itself can be subject to damage. Conversely, deteriorative phenomena, such as corrosion, may cause a tie-rod to lose its functionality and consequently cause the collapse of the structure. Recently, a crack identification method for tie-rods was presented in [23] based on a comparison between the modal parameters of a damaged tie-rod with those of a healthy reference one undergoing the same environmental conditions. The approach mentioned in the study was intended to be adopted through in situ tests, not continuously. Moreover, since the approach requires a comparison with an undamaged twin structure, its adoption in real monitoring applications may be difficult. 

To overcome these limits, the authors of [24] proposed a vibration-based, data-driven approach to tie-rod damage detection that showed the potential to detect damage under the effects of an uncontrolled environment without knowledge of physical variables (e.g., tie-rod tension) and without the need for a reference structure. The key point of the proposed approach is that when a pattern of modal parameters is considered instead of just a single one, the damage-detection problem can be treated as a multivariate outlier detection problem. Previous studies [10,25] have shown that calculating the Mahalanobis squared distance (MSD) of new observations with respect to a baseline period helps mitigate the effects of environmental and operational variations and allows for damage detection. The strategy was validated in an uncontrolled environment to detect damage simulated through the addition of concentrated masses on a tie-rod.

In line with these promising results, three main contributions to the state of the art are made in this paper. First, attention is paid to minimizing the number of required sensors, developing a damage-detection strategy that can be carried out by adopting a single accelerometer on a monitored tie-rod. This strategy relies on a damage feature defined only by tie-rod eigenfrequencies which could be identified with a single accelerometer properly placed on the monitored tie-rod. This allows for a simpler experimental set-up with respect to the one adopted in [24]. The obvious economic consequences of adopting a simple experimental set-up can help the transition from research to real applications.

A second important contribution of this paper is the automatization of the method, so the strategy can be adopted without any human supervision. We present an automatic data-cleansing procedure that was developed and successfully tested on long-term data acquired in an uncontrolled environment. This paper shows how the proposed strategy can significantly improve the performance of a damage-detection strategy based on the MSD.

Finally, the experiment designed to validate the approach represents a one-of-a-kind test case because data referring to the real evolution of a corrosion process over time were considered in this work. This also represents a rare application in the field of structural health monitoring, since the majority of the strategies presented in the literature were only validated with simulated damage in laboratory environment. In this research, instead of producing discrete changes to structural properties (such as the addition of a concentrated mass to simulate a reduction in bending stiffness), real continuous damage evolution was considered over several months, which allowed us to replicate the most realistic and challenging possible scenario to test the effectiveness of our damage-detection strategy for continuous structural health monitoring.

The paper is organized as follows: The adopted vibration-based damage-detection strategy is described, the mathematical background on the MSD is provided, and a damage index is introduced in Section 2. The adoption of an effective data-cleansing algorithm is a necessary step to use the strategy without human supervision, and this algorithm is presented in Section 3. The experimental set-up and the corrosion process that allowed for the introduction of real damage in the monitored tie-rods are described in Section 4. The effectiveness of the damage-detection strategy is discussed in Section 5, where the results of the experimental campaign are presented. The strengths of the method, current limitations, and future developments are discussed in Section 6. Finally, conclusions are drawn in Section 7.

## 2. Damage-Detection Strategy

In this section, the vibration-based damage-detection strategy, in which eigenfrequencies of the monitored structure are used to define a damage feature, is presented. The details related to the extraction of tie-rod eigenfrequencies using a single sensor under operating conditions, together with the data-cleansing procedure proposed to apply the strategy in an uncontrolled environment, are presented in the next section. The discussion presented in this section is generally valid for any structure, regardless of the approach adopted to identify the eigenfrequencies.

If a number M of vibration modes is considered, the M eigenfrequencies $f_{m}$ (with m=1,…,M) can be stored in a feature vector v, defined as follows:

(1) $$v={\left\{f_{1},f_{2},\ldots ,f_{m},\ldots ,f_{M}\right\}}^{T}$$

where the superscript “T” indicates the transpose. More generally, m=1 indicates the first considered eigenfrequency, not necessarily the one associated with the first vibration mode. From a continuous monitoring perspective, the identification of the eigenfrequencies can be repeated a number of times $N_{\mathrm{rec}}$ and the eigenfrequencies can be stored in a matrix of size $N_{\mathrm{rec}}\times M$, as follows:

(2) $$B=\left\{\begin{array}{c} v_{1}^{T}\\ v_{2}^{T}\\ \vdots \\ v_{r}^{T}\\ \vdots \\ v_{N_{\mathrm{rec}}}^{T} \end{array}\right\}$$

with $r=1,\ldots ,N_{\mathrm{rec}}$. 

The matrix B represents a multivariate feature set where every column contains the trend of each of the M considered eigenfrequencies over time. In order to develop an unsupervised learning damage-detection strategy, the behavior of a feature during a reference period (baseline) must be observed and statistically characterized. The matrix containing the eigenfrequencies associated with the baseline period is named $B^{\mathrm{ref}}$ hereafter.

### Damage Index

A new observation of the feature vector during the monitoring period, when the tie-rod health state is unknown, is referred to $v^{\mathrm{new}}$, and multivariate metrics can be adopted to check whether $v^{\mathrm{new}}$ is an outlier with respect to $B^{\mathrm{ref}}$. A damage index can be defined by calculating the MSD between $v^{\mathrm{new}}$ and $B^{\mathrm{ref}}$ according to the following expression:

(3) $$DI=\mathrm{MSD}\left(v^{\mathrm{new}},B^{\mathrm{ref}}\right)={\left(v^{\mathrm{new}}-\mu_{B}^{\mathrm{ref}}\right)}^{T}{\left(\Sigma_{B}^{\mathrm{ref}}\right)}^{-1}\left(v^{\mathrm{new}}-\mu_{B}^{\mathrm{ref}}\right)$$

where $\mu_{B}^{\mathrm{ref}}$ is a column vector of size M×1, the m-th element is the mean of the m-th column of $B^{\mathrm{ref}}$, $\Sigma_{B}^{\mathrm{ref}}$ is the covariance matrix [26] associated with $B^{\mathrm{ref}}$, and superscript “−1” means the inverse. The index DI is the result of a multivariate discordancy test that can be compared against a threshold t to determine whether $v^{\mathrm{new}}$ is judged to be statistically likely or unlikely to have come from the generating process of the multivariate dataset $B^{\mathrm{ref}}$; if DI>t, the new observation $v^{\mathrm{new}}$ is considered to be an outlier with respect to $B^{\mathrm{ref}}$ and damage is detected. The usual condition distribution is assumed to be Gaussian, and the threshold calculation can be carried out in terms of a chi-squared-statistics or by adopting a numerical method. The latter approach based on the Monte Carlo method was adopted in this work following the procedure explained in [27] comprising the following steps:

1. For every iteration, a matrix with the same size of $B^{\mathrm{ref}}$ (i.e., a matrix with $N_{\mathrm{rec}}$ rows and M columns) is considered, where every element is generated from a zero mean and unit standard deviation normal distribution.
2. The MSD is calculated between every row of the matrix and the matrix itself, obtaining $N_{\mathrm{rec}}$ values of DI. The maximum value is stored for every iteration.
3. The procedure is repeated for a large number of trials (e.g., $10^{3}$ times). All the resulted maxima (e.g., $10^{3}$ values) are sorted in terms of magnitude. The critical value for 5% test of discordancy is given by the MSD in the array above which the 5% of the trials occur. In this way, a threshold $t^{*}$—also known as “inclusive threshold”—is obtained. This threshold must be used in cases where the baseline set also contains observations related to the damage condition.
4. If the baseline set does not include data related to the damage condition, as in the considered case, one must adopt another threshold t (also known as “exclusive threshold”) that can be evaluated according to the following expression [1]:
  (4) $$t=\frac{\left(N_{\mathrm{rec}}-1\right){\left(N_{\mathrm{rec}}+1\right)}^{2}t^{*}}{N_{\mathrm{rec}}\left(N_{\mathrm{rec}}^{2}-\left(N_{\mathrm{rec}}+1\right)t^{*}\right)}$$

The threshold level is dependent on both the number of observations ($N_{\mathrm{rec}}$) and the number of variables (M) of the problem being studied. For a given period, the dimensions of the baseline set are dependent on the parameters adopted to obtain a stable automatic identification, as is discussed in the next section.

A key point of the proposed approach is that eigenfrequencies are used to synthetically represent the current state of the monitored tie-rod because representative of all the physical variables that mostly influence its dynamic behavior (e.g., the axial load). Even if these variables change due to environmental and operational variations, the strategy does not require knowledge of them. Indeed, as proven in [25], in order to filter out variability due to environmental and operational conditions, this variability must be included in the samples used to compute the covariance matrix $\Sigma_{B}^{\mathrm{ref}}$. If an adequate baseline set is considered as reference, the MSD becomes almost insensitive to the variations due to environmental effects, and, in this sense, the anomaly detection performance improves when a baseline set containing a full range of environmental conditions is used [10].

A critical aspect of developing a completely automatic damage-detection algorithm is related to the quality of the features used to evaluate $B^{\mathrm{ref}}$ and $v^{\mathrm{new}}$ [28]. Indeed, when the identification of eigenfrequencies is automatically carried out by exploiting the excitation coming from an uncontrolled environment, many possible sources of error can lead to non-reliable results, as discussed in the next section.

If such corrupted data are included in the baseline matrix, they increase the baseline dispersion and make the damage index DI less sensitive to real outlier data related to damage. Moreover, if a vector $v^{\mathrm{new}}$ contains wrong identifications, DI can exceed the threshold when damage is not present. In order to obtain an automatic algorithm that can work in real applications, a data-cleansing procedure was developed to discard features containing wrongly identified eigenfrequencies without the supervision of an expert, as explained in the next section.

## 3. Automatic Identification and Data Cleansing

If the procedure described in Section 2 can be successfully used to detect damage in a tie-rod, as shown in [24], a fundamental step toward real applications is the development of a completely automatic algorithm with no need for human expert supervision. The automatization of the procedure to obtain the data needed by the strategy described in Section 2 is explained below. 

Eigenfrequencies can be automatically identified from the dynamic response of a monitored structure through the adoption of automated operational modal analysis (OMA) [16,29,30] techniques. Among the different possible approaches, a single-degree-of-freedom (SDOF) modal identification technique [31] was adopted in this work to identify the eigenfrequencies of tie-rod bending vibration modes in the vertical plane. Indeed, the monitored tie-rods showed lightly coupled modes that were not closely spaced in frequency and not heavily damped, thus allowing for the adoption of the simple and fast approach described below. However, before discussing the details of the specific case, it is worth mentioning that the data-cleansing procedure and the damage-detection approach proposed here can be carried out regardless of the technique adopted to identify the eigenfrequencies comprising feature vector v. 

When the environment provides random excitation to a tie-rod, each eigenfrequency can be identified through a best fitting between the experimental power spectrum of the response $G_{yy,\exp}\left(\omega \right)$, the function of the angular frequency ω (ω=2πf, where f is the frequency expressed in Hz), and the analytical power spectrum of the response of an SDOF mechanical system with eigenfrequency $f_{m}$ excited by white noise, defined by the following expression [31]:

(5) $$G_{yy,\mathrm{id}}\left(\omega ,f_{m},\zeta_{m},X_{m},A_{m}\right)={\left|\frac{X_{m}}{-\omega^{2}+j2\xi_{m}\left(2\pi f_{m}\right)\omega +{\left(2\pi f_{m}\right)}^{2}}+A_{m}\right|}^{2}$$

where j is the imaginary unit, $\zeta_{m}$ is the *m*-th modal damping ratio, $X_{m}$ is a constant (function of the white noise level, the eigenvector component at the measurement point and the modal participation factor), and $A_{m}$ is the contribution of the out-of-band modes. To allow for more compact notation, these parameters are grouped in a vector $\theta_{m}=\left\{f_{m},\zeta_{m},X_{m},A_{m}\right\}$ such that $G_{yy,\mathrm{id}}\left(\omega ,\theta_{m}\right)$. This simple technique comes with the advantage that a single accelerometer can be adopted if the sensor is placed in a position that is not close to a node of the considered vibration mode.

The Welch’s method, based on the frequency-averaging approach, can be used to calculate an experimental power spectrum [32,33]. The approach requires an initial tuning of some processing parameters that can be set once prior the automatic monitoring. These parameters are related to the duration of the record to analyze T (which determines the amount of time between two observations of the damage feature vector), the duration of the sub-records $T_{\mathrm{sub}}$ used for the averaging procedure (which determines the frequency resolution of the power spectrum $\Delta f=1/T_{\mathrm{sub}}$), the percentage of overlap between two sub-records, and the type of window adopted on every sub-record [34]. The results presented below refer to the considered case study, where a power spectrum was estimated every hour (T=3600 s) using $T_{\mathrm{sub}}=40\;s$, an overlap of 50%, and a Hanning window.

In an uncontrolled environment, the averaging process may not always allow for a good reconstruction of the power spectrum in the frequency bands where the hypothesis of SDOF is made; for this reason, the best fitting procedure may fail or converge to wrong solutions. When this occurs, the wrong estimates of the eigenfrequencies should not be used to define damage feature vectors. We adopted a data-oriented approach that must be seen in the context of continuous monitoring when a huge amount of data are available: identification is always carried out, and wrong identifications are automatically detected and discarded by analyzing the obtained eigenfrequencies. 

The proposed data-cleansing procedure comprises two stages. The first takes place after every eigenfrequency identification, considering vibration modes one at a time, and it is described in Section 3.1. In the second stage of data cleansing (presented in Section 3.2), multiple vibration modes are considered together over a period to detect and remove outliers that are still present after the first stage. 

**Figure 1:**
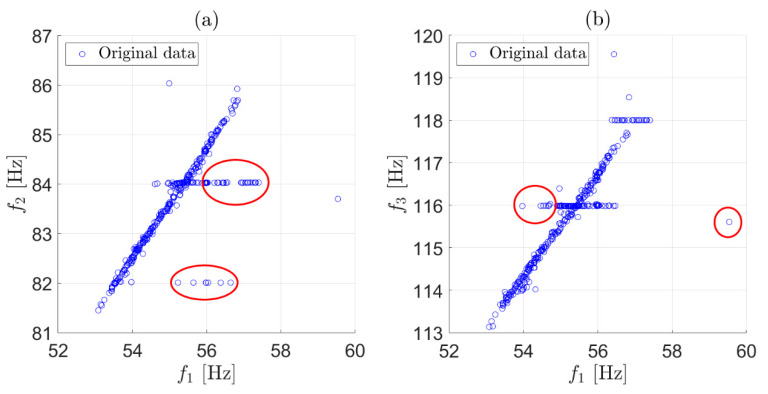
Original data: identified eigenfrequencies during two weeks of monitoring. Scatter plot of $f_{2}$ versus $f_{1}$ (**a**); scatter plot of $f_{3}$ versus $f_{1}$ (**b**).

To show the effect of each stage, we discuss an example based on two weeks of data in which three eigenfrequencies were considered. All the identified frequencies not adopting any data cleansing are presented in Figure 1 as two scatter plots ($f_{2}$ versus $f_{1}$ in Figure 1a and $f_{3}$ versus $f_{1}$ in Figure 1b). The outliers were those observations that significantly deviated from the majority, and they were due to the wrong identification of eigenfrequencies (some examples are circled in Figure 1a,b). 

### 3.1. First Stage

Every m-th eigenfrequency is considered separately every time identification is carried out. The automatic identification procedure considering only one eigenfrequency $f_{m}$ is described below.

**Figure 2:**
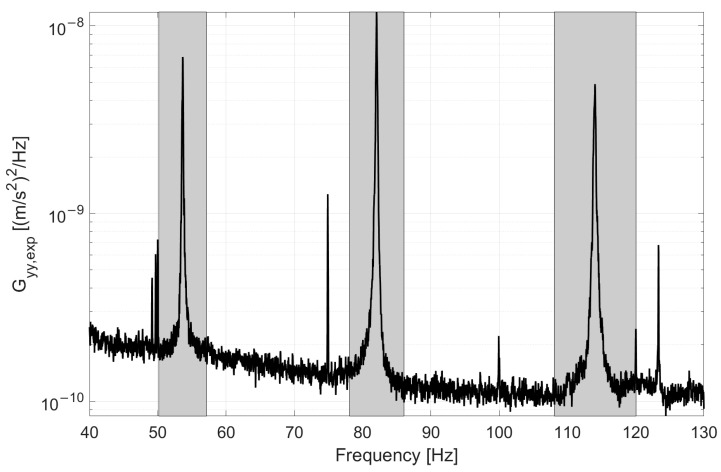
Example of the power spectrum of the response of a tie-rod of the experimental set-up. In grey, the frequency ranges where the SDOF hypothesis is assumed.

1. Initialization step: This step is only intended to define the initial range of where to assume the SDOF hypothesis. The first approximate value of the eigenfrequency $f_{m,0}$ must be indicated, along with a value $\Delta_{m}$, such that the power spectrum is considered only in the range of frequencies between $f_{\min}=f_{m,0}-\frac{\Delta_{m}}{2}$ and $f_{\max}=f_{m,0}+\frac{\Delta_{m}}{2}$. This can be done by roughly identifying the resonance after a visual inspection of the power spectrum at the beginning of the monitoring period. Example initialization parameters used to obtain the eigenfrequencies of the example reported in Figure 1 are presented in Table 1. In this case, these values were defined after a visual inspection of the power spectrum shown in Figure 2, which was obtained from data of duration T=3600 s, $T_{\mathrm{sub}}=40\;s$, an overlap of 50%, and a Hanning window.

2. Assessing the quality of the fitting: When a new record of data of duration T available, the adopted automatic OMA technique is applied to identify the target eigenfrequency. The output of this step is the evaluation of an index that can quantify the quality of the identification. As mentioned above, the best fitting approach was adopted in this work. For this reason, the experimental power spectrum $G_{yy,\exp}$ was first calculated, and only the portion related to the frequencies in the considered range was taken into account. The eigenfrequency $f_{m}$ was estimated by adopting the simplex search method [35] to search for the solution of the minimization problem:

(6) $$\underset{\theta_{m}}{\min}sse$$

where:

(7) $$sse=\overset{i_{\max}}{\underset{i=i_{\min}}{\sum }}{\left(G_{yy,\exp}\left(\omega_{i}\right)-G_{yy,\mathrm{id}}\left(\omega_{i},\theta_{m}\right)\right)}^{2}$$

In Equations (6) and (7), $\omega_{i}=2\pi \left(f_{\min}+i\Delta f\right)$, $i_{\min}=0$ and $i_{\max}=\left(\Delta_{m}/\Delta f\right)$.

If the ideal power spectrum of the response corresponding to the solution of the minimization problem ${\tilde{\theta }}_{m}$ is $G_{yy,\mathrm{id}}\left(\omega ,{\tilde{\theta }}_{m}\right)$, the $R^{2}$ index can be calculated to quantify the quality of the fitting as follows:

(8) $$R^{2}=1-\frac{\sum_{i=i_{\min}}^{i_{\max}}{\left(G_{yy,\mathrm{id}}\left(\omega_{i},{\tilde{\theta }}_{m}\right)-G_{yy,\exp}\left(\omega_{i}\right)\right)}^{2}}{\sum_{i=i_{\min}}^{i_{\max}}{\left(\Gamma -G_{yy,\exp}\left(\omega_{i}\right)\right)}^{2}}$$

where Γ is the mean of $G_{yy,\exp}\left(\omega \right)$ in the considered frequency range. The index $R^{2}$ can be evaluated once the modal parameters are estimated regardless of the adopted identification method.

**Figure 3:**
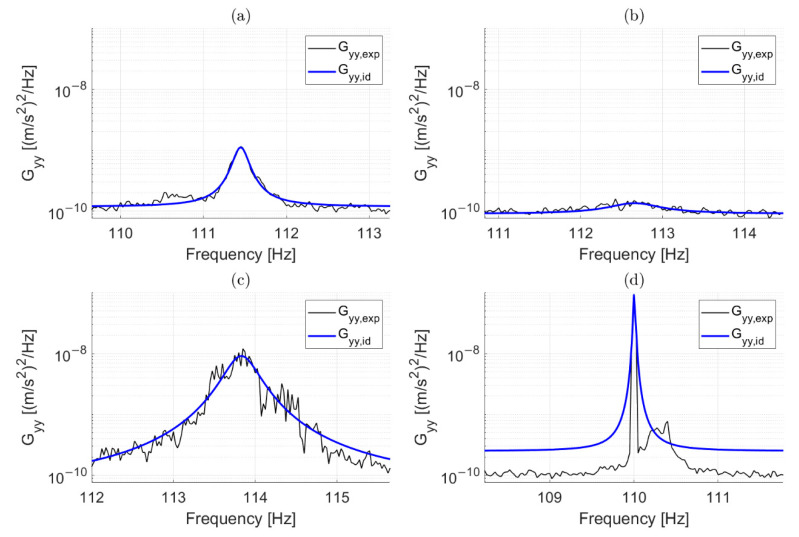
Superimposition of $G_{yy,exp}\left(\omega \right)$ and $G_{yy,id}\left(\omega ,{\tilde{\theta }}_{m}\right)$ on a logarithmic scale for different cases. A successful identification ($R^{2}≅1$ ) (**a**). Lack of excitation of the considered vibration mode ($R^{2}≅0.5$ ) (**b**). Poor signal-to-noise ratio in the considered frequency band ($R^{2}≅0.8$ ) (**c**). Presence of an harmonic input close to the resonance ($R^{2}≅1$ ) (**d**).

Ideally, $R^{2}$ equals 1 (or is very close to 1) if the estimated power spectrum perfectly overlaps with the experimental one. An example is reported in Figure 3a: in this case, the experimental power spectrum (black thin line) and the ideal power spectrum of the response of an SDOF system with an eigenfrequency equal to approximately 111.45 Hz (blue thick line) showed a good match. Lower values of $R^{2}$ are associated with the misidentification of the modal parameters that can occur due to a lack of excitation of the considered vibration mode. An example is presented in Figure 3b. In this case, the vibration mode was not excited, as can be noticed by comparing the amplitude of the experimental power spectrum with that of Figure 3a. In this case, the coefficient $R^{2}$ was approximately equal to 0.5. Other conditions that may be associated with a $R^{2}<1$ are those associated with a poor signal-to-noise ratio that does not allow for an accurate estimate of the power spectrum in the considered frequency range, as can be observed via the shape of the experimental power spectrum presented in Figure 3c. The $R^{2}$ index, in this case, was close to 0.8.

3. Updating $f_{m,0}$ and storing $f_{m}$ in v: According to what previously observed, a threshold level $t_{R}$ can be set to discard wrong identifications. If a $R^{2}\geq t_{R}$ is associated with $f_{m}$, this value is considered as the first guess estimate for the next iteration, so $f_{m,0}$ is updated such that $f_{m,0}=f_{m}$ (and consequently, the considered frequency range is updated). Furthermore, $f_{m}$ is stored in the m-th row of the feature vector v. Conversely, if $R^{2}<t_{R}$, $f_{m,0}$ is not updated and no feature vector v is obtained for the considered record of data. Regardless of the results obtained with the other eigenfrequencies, since the feature vector v must always contain the same number of elements, if even one of the considered eigenfrequencies is not correctly identified, no feature vector v can be obtained for the considered record of data. The procedure iterates starting from step 2.

**Figure 4:**
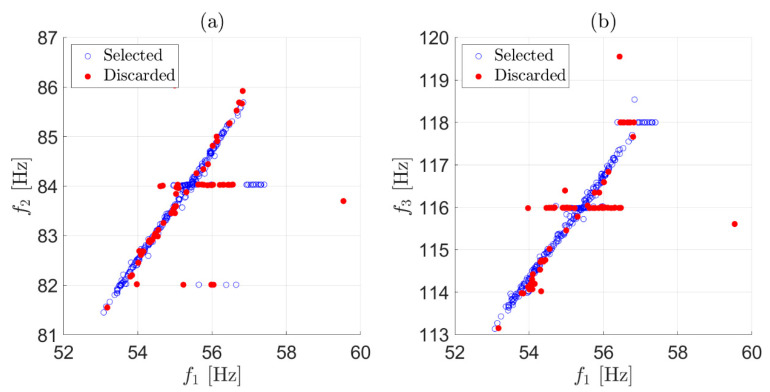
Effect of the first stage of data cleansing on two weeks of data. Scatter plot of $f_{2}$ versus $f_{1}$ (**a**); scatter plot of $f_{3}$ versus $f_{1}$ (**b**). Red-filled circles are the observations discarded by the first stage.

To summarize, at this stage, only eigenfrequency estimates coming from fittings characterized by $R^{2}\geq t_{R}$ are preserved, with decisions made after considering only one record of data at a time and every eigenfrequency separately. This first simple check on the $R^{2}$ value effectively points out the presence of clearly corrupted data (the results shown below were obtained with a threshold level $t_{R}=0.9$). For the example of Figure 1, the strategy was adopted on each of the three eigenfrequencies. The effect of this first stage of data cleansing can be observed in Figure 4, where red-filled circles are associated with identifications that did not satisfy the condition $R^{2}\geq 0.9$ and were discarded. Although a number of outliers were correctly detected and removed by the first stage, there were still observations that deviated from the majority of the population, meaning that some wrong eigenfrequency identifications could still be associated with a high $R^{2}$. An example related to the presence of a harmonic disturbance is presented in Figure 3d: in this case, the solution provided by the simplex search method is the value of the harmonic disturbance at 110 Hz, underestimating the correct eigenfrequency value for the considered vibration mode and still resulting in an $R^{2}≅1$. In general, to remove outliers still present after the check on $R^{2}$, another stage of data cleansing is needed.

### 3.2. Second Stage

This time, multiple observations of the feature vector v (i.e., matrixes B) are considered. The second strategy for outlier removal considers the trend in time of the eigenfrequencies over a short-term period, assuming that in such a short-time window, eigenfrequencies variations are only caused by axial load variations, like those caused by temperature variations, and not by damage. This assumption is valid when considering long-term deteriorative phenomena that do not significantly evolve in a short period, e.g., one or two weeks.

From analytical models presented in the literature [36,37], every tie-rod squared eigenfrequency is linearly dependent on the axial load. Consequently, squared eigenfrequencies of different vibration modes are related to each other with a linear relationship if the axial load is the only changing variable. 

If a set $B^{\mathrm{short}}$ is considered, the columns of the matrix are the trends of the M identified eigenfrequencies in a short-term period, and they can be indicated as vectors $e_{m}$, with m=1,…M. All the elements in vectors $e_{m}$ can be squared, and the resulting vectors are indicated as $s_{m}$ hereafter, with m=1,…,M. As an example, two vectors $s_{i}$ and $s_{j}$ are considered now, with i<j: if the only changing variable is the axial load, a scatter plot showing the values of $s_{j}$ as a function of the values of $s_{i}$ should result in points lying on a line (or scattered around a line when dispersion due to identification uncertainty is considered). Conversely, abnormal observations that cannot be explained by the assumed underlying linear model would deviate from the linear trend. 

To exploit this idea in an automatic way, all the M−1 couples made by the lowest squared eigenfrequency and one of the other M−1 squared eigenfrequencies are considered separately (in the discussed example where three eigenfrequencies $f_{1}$, $f_{2}$ and $f_{3}$ are considered, two couples can be made: $s_{1}$-$s_{2}$ and $s_{1}$-$s_{3}$). For every couple, the linear trend is first estimated by only considering a subset of observations, as explained in Section 3.2.1. Once the linear trend is characterized, data are fitted to the line and observations that show a high residual are removed, as explained in Section 3.2.2.

#### 3.2.1. Linear Trend Estimate

First, the linear trend between $s_{i}$ and $s_{j}$ must be estimated over a short-term period (two weeks of data in the considered example). To estimate the coefficients of the linear trend, it is important to consider that, at this stage, outliers may be present in one or both vectors. Evaluating the coefficients of the linear trend while also considering these outliers can lead to biased results, so the identification of the linear trend is carried out only on a sub-set of observations. A pre-selection of data is carried out using the Hampel Identifier [28] on each of the two vectors. The Hampel Identifier is a variation of the three-sigma rule of statistics that is robust against outliers. When data contain outliers, even a single out-of-scale observation can cause significant changes of the sample mean and variance. For this reason, the median and the median absolute deviation (MAD) are used to estimate the data mean and standard deviation, respectively. 

**Figure 5:**
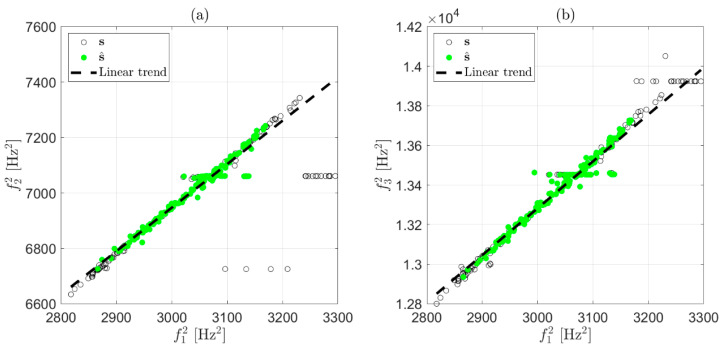
Linear trend estimate considering a subset $\hat{s}$ (in green-filled circles) of the available observations s (black circles). Scatter plot of $f_{2}^{2}$ versus $f_{1}^{2}$ (**a**); scatter plot of $f_{3}^{2}$ versus $f_{1}^{2}$ (**b**).

Only data of $s_{i}$ and $s_{j}$ that are less than 1 scaled MAD (see Appendix A) distant from the local median over a moving window of 72 h (green-filled circles in Figure 5) are considered to estimate linear trend coefficients. The vectors that contain the data used to carry out a linear fit are indicated with the symbols ${\hat{s}}_{i}$ and ${\hat{s}}_{j}$. The coefficients of the linear regression $a_{ij}$ and $b_{ij}$ are those coming from the least squares solution of the linear problem:

(9) $${\hat{s}}_{j}=a_{ij}\cdot {\hat{s}}_{i}+b_{ij}\cdot u$$

where u is a column vector of the same size as ${\hat{s}}_{i}$ or ${\hat{s}}_{j}$, with all elements equal to 1.

In the example, the two couples of squared eigenfrequencies resulting from the first stage of the data-cleansing strategy are reported in Figure 5 (all the black and green-filled circles). The observations corresponding to green-filled circles were those considered to calculate $a_{12}$ and $b_{12}$ (i.e., the coefficients of the black-dashed line in Figure 5a) and $a_{13}$ and $b_{13}$ (i.e., the coefficients of the black-dashed line in Figure 5b).

#### 3.2.2. Discard with Residual

Once the coefficients $a_{ij}$ and $b_{ij}$ are known, vectors $s_{i}$ and $s_{j}$ are considered at this stage to finally carry out outlier removal based on the residuals $\epsilon_{ij}$ of the linear fitting:

(10) $$\epsilon_{ij}=s_{j}-a_{ij}\cdot s_{i}-b_{ij}\cdot u$$

**Figure 6:**
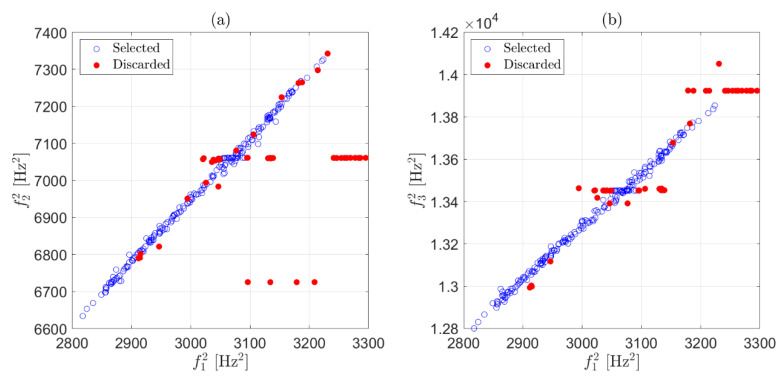
Effect of the second stage of data cleansing on two weeks of data. Scatter plot of $s_{2}$ versus $s_{1}$ (**a**); scatter plot of $s_{3}$ versus $s_{1}$ (**b**). Red-filled circles are the observations discarded by the second stage.

The median and the scaled MAD of the elements of vector $\epsilon_{ij}$ are calculated. Every element that is more than 2 scaled MAD away from the median is considered an outlier and the corresponding row of $B^{\mathrm{short}}$ is removed. This check can be carried out on a moving window of 72 h, as in the previous step, but it is also possible to use a broader window. In the case, as an example, the outliers identified with a two-week window are those reported with red-filled circles in Figure 6. Similarly to what discussed in Section 3.1, when the check on either $\epsilon_{12}$ or $\epsilon_{13}$ marks an observation as an outlier, the corresponding three eigenfrequency estimates $f_{1},f_{2}$ and $f_{3}$ are discarded. For this reason, when an outlier was detected, e.g., because of the check on $\epsilon_{12}$, the corresponding observation is marked with a red filled circle in Figure 6a,b.

**Figure 7:**
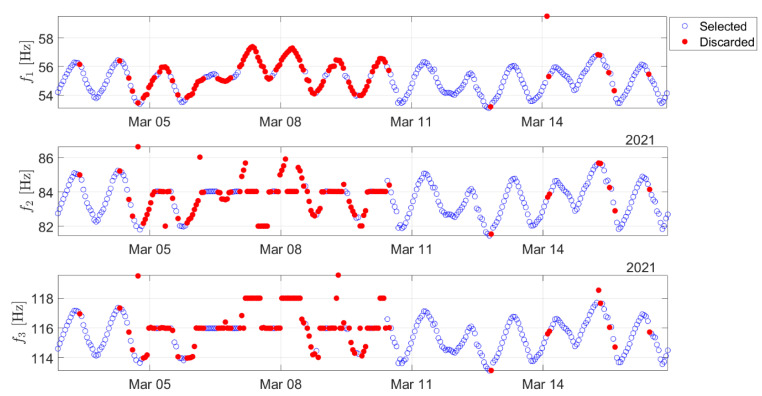
Trend in time of the identified eigenfrequencies during two weeks of monitoring: red-filled circles indicate the observations that were automatically discarded by the data-cleansing procedure.

In conclusion, the total effect of the proposed strategy on the previous example can be observed in Figure 7, where the eigenfrequencies selected and those discarded by the automatic data-cleansing procedure are indicated with blue circles and red-filled circles, respectively. As shown in Section 5, the adoption of the data-cleansing strategy allowed the automatic application of the algorithm introduced in Section 2 to successfully detect real damage in an uncontrolled environment. Before discussing the results, the test case is presented in the next section.

## 4. Experimental Set-Up

**Figure 8:**
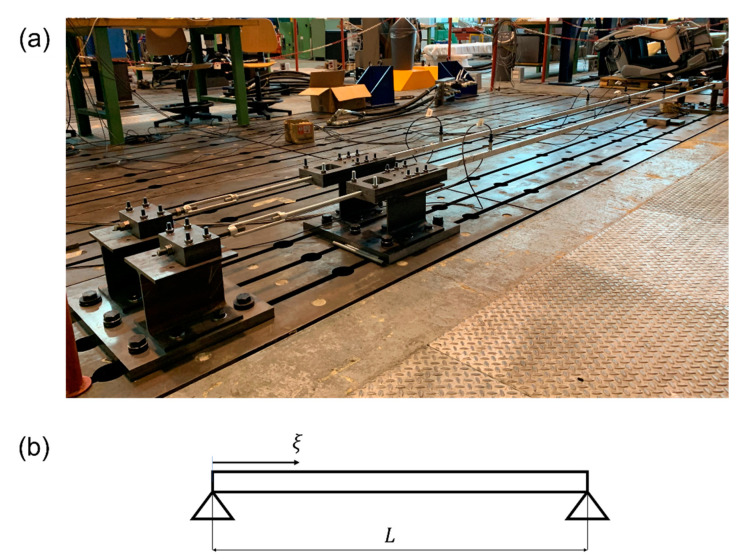
(**a**) The experimental set-up; (**b**) convention for coordinate ξ.

The experimental set-up (see Figure 8a) comprised two nominally identical full-scale aluminum tie-rods with a free length of 4 m and a cross-section of $0.015\times 0.025\;m^{2}$. The set-up was located in the laboratories of Politecnico di Milano, specifically in a room where numerous activities (mainly human activities and those related to laboratory testing machines) take place throughout the day. Furthermore, the temperature is intentionally not controlled, so environmental and operational variations are those of an uncontrolled environment. More specifically, throughout the monitoring period, the maximum and minimum observed laboratory temperatures were approximately 6 and 29 °C, respectively, with daily thermal excursion from 3 to 8 °C.

The tie-rods were equipped with sensors to replicate a long-term structural health monitoring system for research purposes (e.g., [24,38]). More specifically, each tie-rod was equipped with four general-purpose industrial piezo-electric accelerometers (PCB 603C01 model with a sensitivity of 10.2 mV/(m/s^2^) and full scale of ±490 m/s^2^). The choice for general-purpose industrial accelerometers comes from the decision to not adopt high-end sensors, which are typical of laboratory environments and not representative of real applications. Furthermore, strain gauges comprising a calibrated full Wheatstone bridge were used to measure the axial load, and the laboratory temperature was measured with a thermocouple close to the tie-rods. 

Data were acquired with NI9234 modules with anti-aliasing filter on board at a sampling frequency of 512 Hz, obtaining a bandwidth of approximately 200 Hz that included the range of frequency significantly excited by the operative environment. After some preliminary tests, it was observed that under normal conditions, the operating environment usually provided a broadband excitation that significantly decreased above 200 Hz.

**Figure 9:**
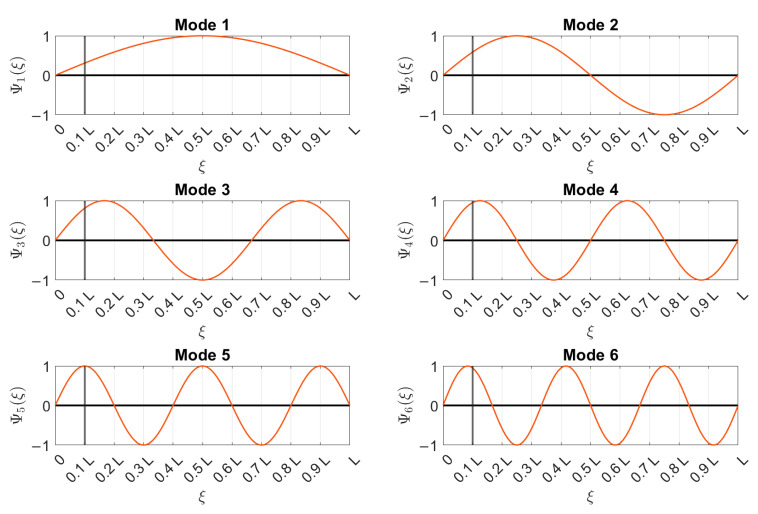
Analytical mode shapes and positions of the accelerometer, as indicated by a vertical black-solid line.

The data presented below were acquired with an accelerometer placed at $\xi =\frac{L}{10}$ (L is the beam free-length and ξ is the longitudinal distance from the constraint according to the scheme in Figure 8b). In order to adopt the proposed damage-detection strategy using only one sensor, the accelerometer position was selected while trying to avoid a vibration node of the first six bending vibration modes in the vertical plane. Figure 9 shows the analytical mode shapes of the six considered modes for a pinned–pinned beam subject to axial load (the simple pinned–pinned model was used here to show the concept behind the accelerometer position selection). The coordinate ξ is reported on the *x*-axis, and every m-th mode shape $\Psi_{m}\left(\xi \right)$, with m=1,…,M and normalized to one, is presented on the *y*-axis. The position of the accelerometer is indicated by a black-solid vertical line at ξ=L/10: it is possible to observe that such a position potentially allowed for the identification of the considered vibration modes.

As a general rule of thumb, the position of the accelerometer must be chosen after considering how many vibration modes are identifiable given the operational environment. When the number of modes is known, the accelerometer must be placed as far as possible from the constraints in a position that is not a vibration node for the considered modes. This choice is important to correctly identify the eigenfrequencies from structural dynamic responses. In practical cases, even though the mode shapes of a real tensioned beam are not exactly coincident with those of simplified analytical models, the positions of modal nodes do not significantly differ and can thus be avoided. Alternatively, for a more accurate selection of the position of the sensor, one can consider carrying out a preliminary experimental campaign at the beginning of the monitoring aimed at identifying the mode shapes. In this case, OMA can be carried out by using an adequate number of accelerometers; once the vibration modes are reconstructed, the single sensor used for long-term monitoring can be placed while avoiding modal nodes. Moreover, since multiple possible options are available, the choice must fall on the position that can provide the best signal-to-noise ratio (e.g., far from the constraints, where the eigenvector components are generally low). A better quality of vibration data in terms of the signal-to-noise ratio increases the reliability of the automatic identification process, which has an impact on the proposed damage-detection algorithm performance.

For our experiment, vibration data were continuously acquired under the effect of uncontrolled environmental and operational variations throughout approximately one year, first to define the baseline set and then to monitor the evolution of real damage during the evolution of a corrosion process, as described in the next sub-section.

### The Corrosion Process

A key part of the experiment was the introduction of real damage to the tie-rods, which represents a one-of-a-kind application in the literature of tie-rod damage detection and, more generally, structural health monitoring. A corrosion process was started on one of the two tie-rods. The type of corrosion attack reproduced in the experiment is referred to as “general corrosion”, where the electrochemical reactions between the metal and the chemical to which it is exposed cause a uniform loss of the metal thickness over the entire exposed surface. Although aluminum is a chemically very reactive metal, its behavior is made stable due to the formation of a protective adherent oxide film on the surface. This film is generated in a natural way, and it is immediately reproduced in the presence of oxygen, thus protecting the substrate from further oxidation phenomena. Only when the natural protection provided by the oxide is destroyed under the action of chemical agents and its regeneration is inhibited can corrosion occur in its various forms [39]. 

**Figure 10:**
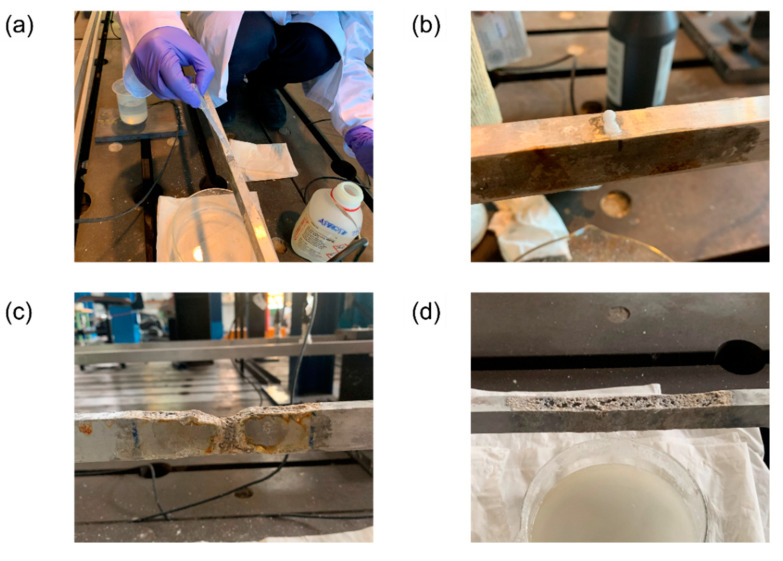
(**a**) application of HF to the top of a tie-rod; (**b**) application of NaOH to the top of a tie-rod; (**c**) the result of the corrosion process at ξ=L/10 on 25 May 2021; (**d**) the result of the corrosion process at $\xi =\frac{5}{8}L$ on 25 May 2021.

The natural film can be attacked and dissolved both by strongly alkaline solutions and an acid pH; the most sever attacks have been recorded in the presence of concentrated solutions of sodium hydroxide (NaOH) and hydrofluoric acids (HF) [39]. In line with this observation, a general corrosion process was induced on a portion of each of the two tie-rods by alternating the direct application of HF (Figure 10a) and NaOH (Figure 10b) on the top surface. First, HF was used to dissolve the protective film so that the general corrosion process could start more easily. Then, a highly concentrated solution of NaOH and water was applied to corrode the portion of the tie-rod. The reaction did not self-feed, and once the NaOH stopped corroding, the formation of the protective oxide resumed. Moreover, since the corrosion products of NaOH helped the formation of the oxide, they were brushed away from the surface of the tie-rods, and the procedure was periodically repeated, starting again from the application of HF.

One of the two tie-rods was attacked for the first time on 8 November 2020 at $\xi =\frac{9}{10}L$, thus introducing damage close to the constraints. The extent of the area subject to general corrosion was approximately 5 cm, and the final appearance of the tie-rod on 25 May 2021 is presented in Figure 10c. On 23 February 2021, the same procedure started on the second tie-rod at $\xi =\frac{5}{8}L$, for an extension of 10 cm. The final aspect of the tie-rod on 25 May 2021 can be observed in Figure 10d. 

## 5. Results

**Figure 11:**
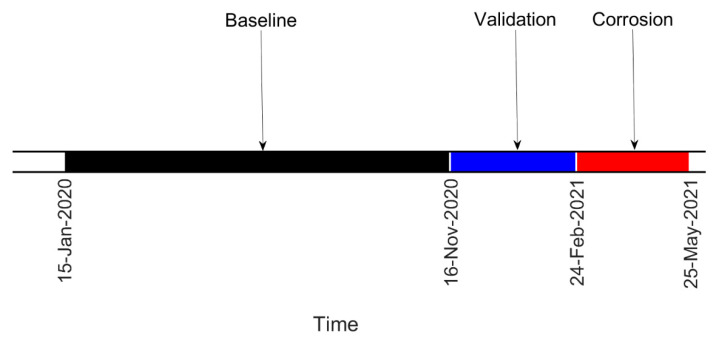
Timeline of the test on the tie-rod corroded at $\xi =\frac{5}{8}L$.

In this section, the results of the experimental campaign are presented. First, damage farther from the constraints is considered, so the results refer to the tie-rod corroded at $\xi =\frac{5}{8}L$. In Figure 11, the timeline of the experiment is reported. The acquired data can be divided into three sets: baseline, validation and corrosion. Data belonging to the baseline set were those used to build the matrix $B^{\mathrm{ref}}$, which was the reference for the calculation of the damage index DI through the MSD (see Section 2). The validation set contained data acquired when damage was not present on the tie-rod but that were not included in the matrix $B^{\mathrm{ref}}$; this set was used to check that the damage index did not exceed the threshold level t when damage was not present, causing false alarms. Finally, the corrosion set contained data referring to the period when the chemical attack was ongoing and damage was progressing. 

**Figure 12:**
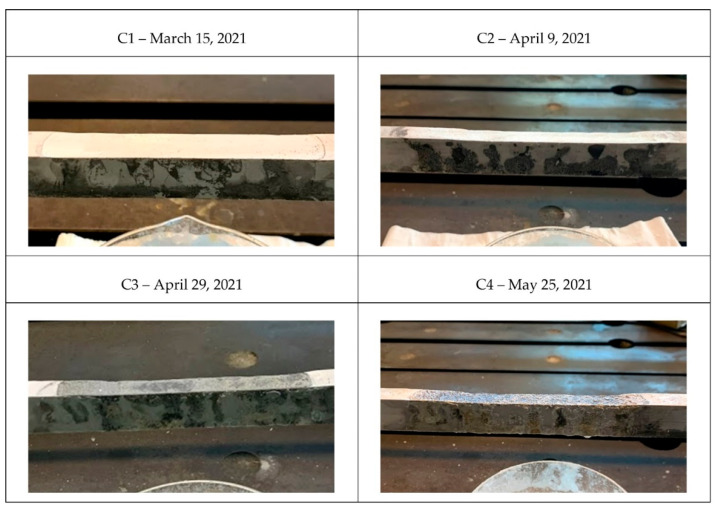
Different corrosion conditions for damage farther from the constraints.

**Figure 13:**
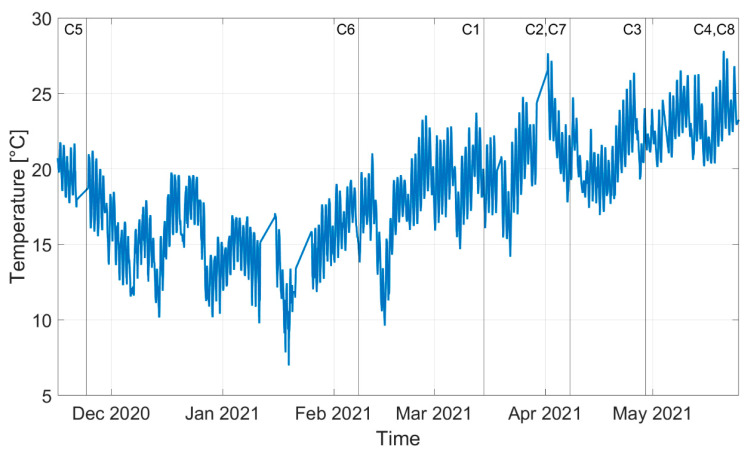
Trend in time of the laboratory temperature measured with a thermocouple located in the proximity of the monitored tie-rods.

**Figure 14:**
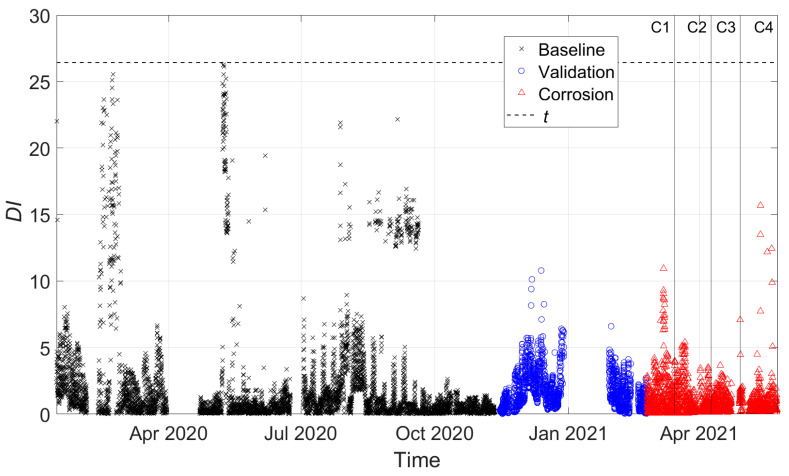
DI for damage farther from to the constraints (no data cleansing adopted).

**Figure 15:**
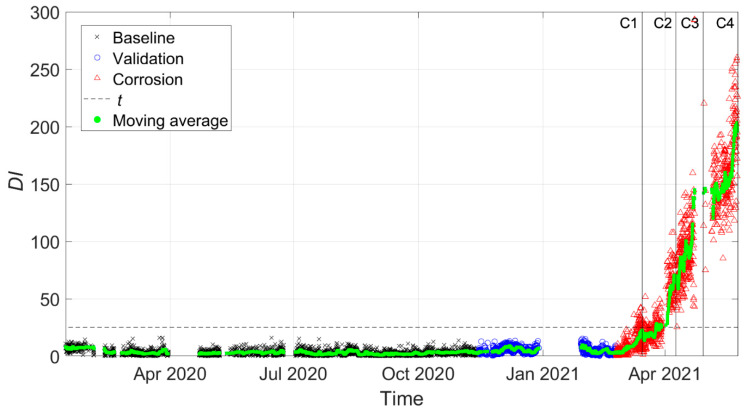
DI for damage farther from to the constraints (data cleansing adopted).

Four pictures of different states of the tie-rod during the corrosion process with the respective dates are presented in Figure 12: labels C1, C2, C3 and C4 are used in Figure 13, Figure 14 and Figure 15 to enable direct references to the tie-rod condition.

Moreover, the magnitude of damage was quantified by measuring the reduction in the height of the tie-rod cross section at the center of the corroded area. In Table 2, the percentage of reduction in the height of the cross section with respect to the initial condition, indicated by the symbol Δh, is reported for every damage condition (conditions C5, C6, C7, and C8 are related to the case of damage close to the constraints, as discussed below).

The different temperature conditions corresponding to the different corrosion conditions can distinguished from the trend of the laboratory temperature over time presented in Figure 13, where different vertical lines with markers refer to different stages of the corrosion process shown in Figure 12 (in this case, labels C5, C6, C7 and C8 refer to the case of damage close to the constraints, as described below).

Figure 14 and Figure 15 present the damage index DI, which was calculated between every observation of the feature vector and $B^{\mathrm{ref}}$ considering three tie-rod eigenfrequencies, more specifically those of the third, fourth and fifth vibration modes. In the following figures, black crosses indicate DI calculated on baseline data, blue circles indicate DI calculated on the validation set, and red triangles indicate DI calculated during the corrosion process. Different vertical lines with markers refer to different stages of the corrosion process according to Figure 12. Finally, the horizontal black-dashed line represents the threshold level t for the MSD-based outlier detection (see Section 2).

If the automatic procedure does not include any data-cleansing process, different problems arise, as shown in Figure 14. Due to the presence of a high number of wrong eigenfrequency identifications, the scatter of the baseline data was high. This caused the method to be less sensitive to damage, which was not detected (red triangles are always significantly below the damage threshold). Moreover, no trend related to an evolving state of damage could be assessed if red triangles were considered and compared with baseline (black crosses) or validation data (blue circles).

The results presented in Figure 15 were obtained with data that were automatically selected by the data-cleansing procedure. Since the baseline set, by definition, only included data acquired when the health state of the tie-rod was known and damage was not present, the data-cleansing process explained in Section 3.2 was conducted while considering all the observations in the baseline set. For the other two sets (Validation and Corrosion), the procedure was instead carried out with two weeks of data at a time because the structural properties of the tie-rod could have changed due to possible damage and evolution.

It is possible to observe that the situation was significantly improved: all the black crosses fall below the threshold, in accordance with the fact that no outlier related to damage was present in the baseline set. Furthermore, the blue circles related to the validation set do not fall above the threshold level, thus not causing false positives. During the corrosion process, the index values represented by red triangles deviated from the range of black crosses and blue circles, showing an increasing trend that can be more easily assessed by looking at a moving average trend reported in green (obtained with a moving average window of duration equal to one day that was shifted every hour). 

When the moving average first crossed the threshold, the tie-rod was not yet in the condition C2, so Δh was between 2% and 5%. The result is remarkable considering that the damage state C2 (Δh=5%) was barely visible during a visual inspection of the tie-rod, with reference to the picture in Figure 12.

The performance of the automatic damage-detection algorithm can be represented through the adoption of a receiver operating characteristic (ROC) curve [40]. This graphical tool is widely adopted to illustrate the capability of a binary classifier to detect damage as a threshold is varied. An ROC is made by plotting the true positive rate (TPR) against the false positive rate (FPR) at various threshold levels. The TPR is the ratio between the number of positives correctly identified as positives (number of red triangles above the threshold) and the total number of positives (total number of red triangles). The FPR is the ratio between the number of false positives (number of blue circles above the threshold) and the number of negatives (total number of the blue circles). A perfect classifier ROC is composed by two straight lines from the origin with coordinates (0,0) to the top left corner (0,1) and from (0,1) to the top right corner (1,1), while a random classifier is represented by a diagonal from (0,0) to (1,1). The resulting plot can be used to compare the relative performance of different classifiers and to determine whether a classifier performs better than random guessing.

**Figure 16:**
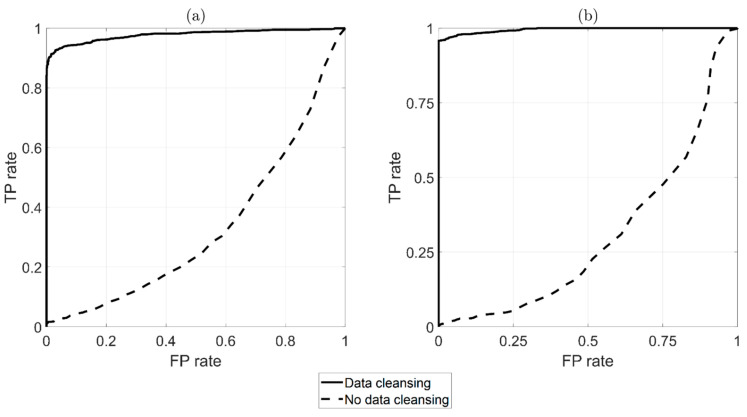
ROC comparison between adopting (black-solid line) or non-adopting (black-dashed line) the automatic data-cleansing procedure prior to damage detection (damage at $\xi =\frac{5}{8}L$). Results are presented without (**a**) and with (**b**) a moving average.

Figure 16 shows a comparison of the ROC curves with and without the automatic data-cleansing procedure, as indicated by black-solid and black-dashed lines, respectively. Figure 16a was derived from DI calculated every hour, while Figure 16b as derived from data obtained with the moving average. It is possible to observe that the data-cleansing algorithm was fundamental for the strategy to be automatically adopted for damage detection (compare the black-dashed line with the black-solid line in Figure 16a,b). Indeed, the black-dashed lines indicate that the damage-detection algorithm’s performance was worse than that of a random classifier; conversely, the black-solid lines indicate behavior that was very close to that of a perfect classifier. 

**Figure 17:**
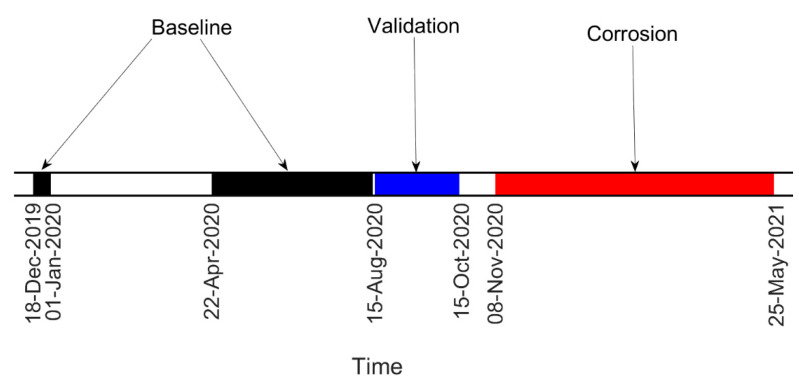
Timeline of the test on the tie-rod corroded at *ξ* = 9/10 L.

The same conclusions can be drawn for the other tie-rod experiment that considered damage close to the constraints (damage at $\xi =\frac{9}{10}L$). In this case, the timeline of the experiment is reported in Figure 17, again adopting the same labels previously used to identify the different datasets. 

**Figure 18:**
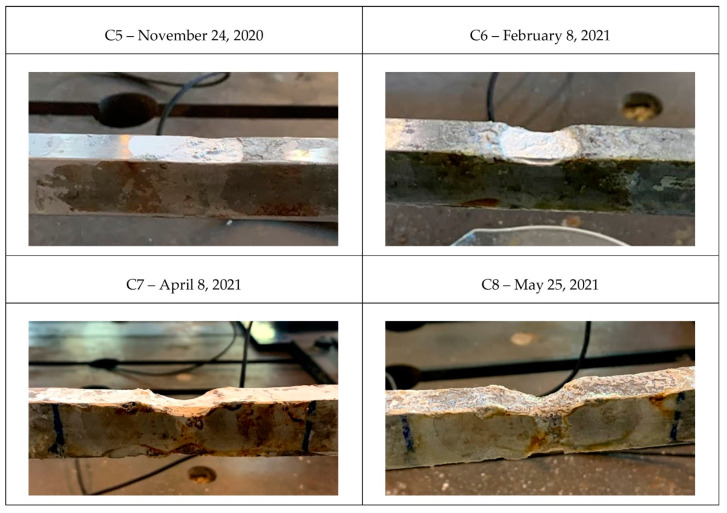
Different corrosion conditions for damage close to the constraints.

**Figure 19:**
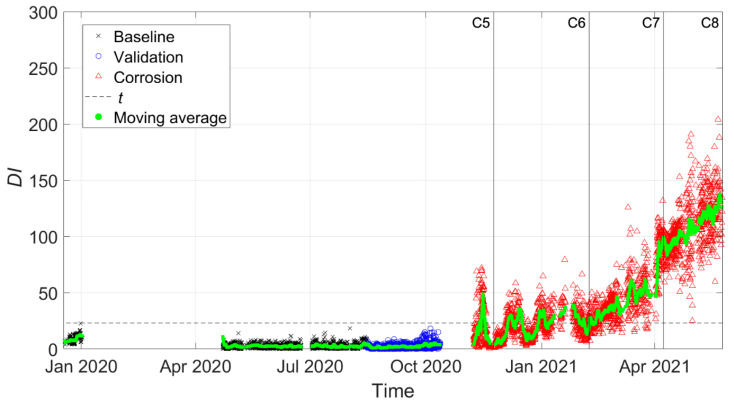
DI for damage close to the constraints (data cleansing adopted).

As in the previous case, different states of the corrosion process are presented in Figure 18. The labels adopted in Figure 19, where DI is presented for the second tie-rod, are the same as those reported in Figure 18. The severity of the damage in the different conditions is quantified by Δh in Table 2, while the temperature conditions are shown in Figure 13. The damage index DI was evaluated while considering three tie-rod eigenfrequencies, more specifically those of the fourth, fifth and sixth vibration modes.

**Figure 20:**
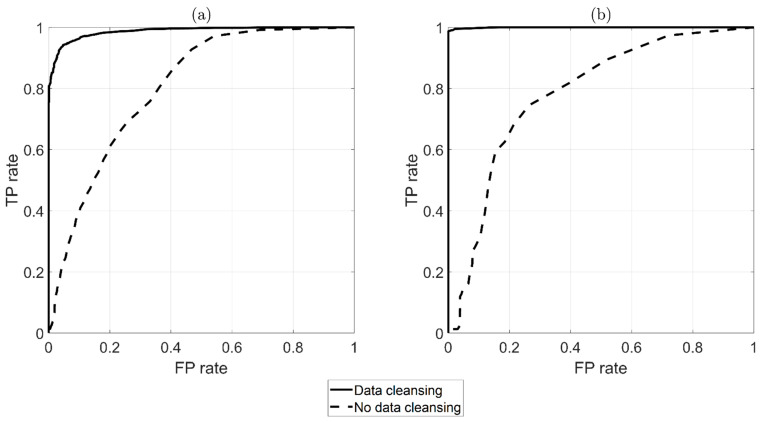
ROC comparison between adopting (black-solid line) and not adopting (black-dashed line) the automatic data-cleansing procedure prior to damage detection (damage at $\xi =\frac{9}{10}L$). Results are presented without (**a**) and with (**b**) a moving average.

Additionally in this case, the strategy could successfully detect damage, and the results were remarkable considering the extent and severity of the damage in a location close to the constraints where the index is less sensitive to damage, as shown in [24]. Moreover, it is worth noticing that, in this case, the baseline set was shorter than that of damage at $\xi =\frac{5}{8}L$ (compare timelines reported in Figure 11 and Figure 17) due to the fact that the corrosion attack started earlier and that the tie-rod was used for another experimental test between 1 January 2020 and 22 April 2020. Since the performance of MSD-based damage detection improves when a large-enough baseline set is adopted to capture a full range of environmental conditions [10,25], the results presented in Figure 19 could improve if a larger baseline set was adopted. Finally the ROC curves for this case are presented in Figure 20: again, it is possible to observe how the performance of the damage-detection algorithm was improved by the adoption of the data-cleansing procedure.

## 6. Strengths of the Method, Current Limitations and Future Developments

In summary, the proposed method allows for tie-rod monitoring with a single sensor through an easy-to-apply algorithm that does not require knowledge of physical variables, e.g., the tie-rod axial load. By applying the proposed data-cleansing procedure, the damage-detection algorithm based on the MSD can be adopted in a completely automatic way. As proven by the results of the experimental campaign, the presented strategy allows for the successful detection of real damage under the effect of uncontrolled operational and environmental variations.

In practice, since the proposed approach requires the estimate of the eigenfrequencies of multiple vibration modes, its main limitation is related to the frequency band of the environmental disturbance that may sometimes limit the number of eigenfrequencies that can be correctly identified. Future research may regard the improvement of the sensitivity of the method and the evaluation of the damage-detection performance when other types of damage are considered.

## 7. Conclusions

An automatic, vibration-based, data-driven damage-detection algorithm for beam-like structures is proposed in this work. The approach allows for damage detection with the use of a single accelerometer on a monitored tie-rod, does not require time-based inspections, and can be carried out without the supervision of a human operator. 

The relationship between the eigenfrequency values of different vibration modes is exploited to discard corrupted data at the data-cleansing stage and to detect damage through the adoption of the MSD. The potential of the strategy in real applications was experimentally proven, as real damage to full-scale tie-rods was detected both close to and farther from the constraints under the effect of an intentionally uncontrolled environment. Testing the algorithm under such a challenging and realistic scenario has allowed us to show the potential of the proposed strategy to be successfully translated to real applications.

## Figures and Tables

**Table 1:** Example of initialization parameters referring to the power spectrum in Figure 2.

m	$f_{m,0}\;(\mathrm{Hz})$	$\Delta_{m}\;(\mathrm{Hz})$
1	53	7
2	82	8
3	114	12

**Table 2:** Different magnitude of damage expressed in terms of Δh.

Corrosion Condition	Δh (%)
C1	2
C2	5
C3	6
C4	10
C5	6
C6	8
C7	22
C8	28

## Data Availability

The data presented in this study are available on request from the corresponding author.

## Appendix A

A vector h, with dimensions (N×1), is considered here. The r-th element of h is indicated with $h_{r}$. To robustly estimate the mean and the scatter of h, the median and the MAD are often recommended. The MAD is defined as follows:

(A1) $$\mathrm{MAD}\left(h\right)=\mathrm{median}\left(\left\{\left|h_{1}-\mathrm{median}\left(h\right)\right|,\left|h_{2}-\mathrm{median}\left(h\right)\right|,\ldots ,\left|h_{r}-\mathrm{median}\left(h\right)\right|,\ldots ,\left|h_{N}-\mathrm{median}\left(h\right)\right|\right\}\right)$$

According to [41], the element $h_{r}\;$is considered an outlier for a given threshold $n_{\sigma }$ if:

(A2) $$\left|h_{r}-\mathrm{median}\left(h\right)\right|\geq n_{\sigma }\cdot \kappa \cdot \mathrm{MAD}\left(h\right)$$

The quantity κ·MAD(h) is called scaled MAD, and κ is a normalization factor equal to 1.4826 [42] that was chosen to make the expected value of the scaled MAD equal to the standard deviation if h contains a Gaussian data sequence [42].

In this work, $n_{\sigma }=1$ on a moving window of 72 h was adopted on every single vector s to select the data that comprised vectors $\hat{s}$, i.e., those used for the linear trend coefficient estimates (see Section 3.2.1). $n_{\sigma }=2$ was adopted to identify the outliers in vectors $\epsilon_{ij}$ (see Section 3.2.2) and finally discard the corrupted identifications.
